# Reciprocal Modulation of Tumour and Immune Cell Motility: Uncovering Dynamic Interplays and Therapeutic Approaches

**DOI:** 10.3390/cancers17091547

**Published:** 2025-05-01

**Authors:** Angelo Aquino, Ornella Franzese

**Affiliations:** Department of Systems Medicine, University of Rome Tor Vergata, Via Montpellier 1, 00133 Rome, Italy; angelo.aquino@uniroma2.it

**Keywords:** cell motility, tumour microenvironment (TME), epithelial mesenchymal transition (EMT), T-cells, chemokines, therapeutic target, resistance, immune checkpoint blockade

## Abstract

Dysregulated cell movement is a hallmark of cancer progression and metastasis, the leading cause of cancer-related mortality. The metastatic cascade involves tumour cell migration, invasion, intravasation, dissemination, and colonisation of distant organs. These processes are influenced by reciprocal interactions between cancer cells and the tumour microenvironment (TME), including immune cells, stromal components, and extracellular matrix proteins. The epithelial–mesenchymal transition (EMT) plays a crucial role in providing cancer cells with invasive and stem-like properties, promoting dissemination and resistance to apoptosis. Conversely, the mesenchymal–epithelial transition (MET) facilitates metastatic colonisation and tumour re-initiation. Immune cells within the TME contribute to either anti-tumour response or immune evasion. These cells secrete cytokines, chemokines, and growth factors that shape the immune landscape and influence responses to immunotherapy. Notably, immune checkpoint blockade (ICB) has transformed cancer treatment, yet its efficacy is often dictated by the immune composition of the tumour site. Elucidating the molecular cross-talk between immune and cancer cells, identifying predictive biomarkers for ICB response, and developing strategies to convert cold tumours into immune-active environments is critical to overcoming resistance to immunotherapy and improving patient survival.

## Simple Summary

In this review, we describe the key mechanisms controlling tumour and immune cell motility within the tumour microenvironment and how these dynamics affect cancer progression, metastasis, and response to therapy. We focus on how tumours can hinder T-cell access promoting immune-excluded phenotypes. At the same time, regulatory immune cells such as tumour-associated macrophages (TAMs) and regulatory T-cells (Tregs) can enhance tumour cell migration and invasion by promoting processes like epithelial–mesenchymal transition (EMT) and producing signals that support tumour cell migration. These processes create an environment that impairs the immune response against the tumour. Understanding these mechanisms can help identify biomarkers predicting patients’ response to immunotherapy and support the development of treatments that make tumours more accessible to immune cells, improving therapeutic outcomes.

## 1. Unravelling the Mechanisms Regulating Tumour Cell Motility

### 1.1. An Overview

Cell motility is a fundamental and tightly regulated process exhibited by all cells. It plays a key role in various biological contexts, including embryonic development, morphogenesis, organogenesis, tissue remodelling, wound healing, immune responses, angiogenesis, tissue repair, tissue regeneration, and cell differentiation. Furthermore, cell movement is also critically involved in a wide range of human pathological conditions such as cancer development, progression, and metastasis [1].

Although numerous studies have investigated the molecular and biochemical mechanisms underlying the motility of individual cells, the broader principles regulating cell motility at the systemic level remain poorly understood.

The processes of migration, local tissue invasion, and distant metastatic spread are complex biological mechanisms that significantly worsen patient outcomes and represent the primary drivers of cancer-related mortality [2,3].

During tumour initiation, progression, and metastatic spread, dynamic and reciprocal cross-talk between cancer cells and their surrounding microenvironment reprograms cellular behaviour through extensive molecular and phenotypic alterations [4,5].

The tumour microenvironment (TME) and epithelial–mesenchymal transition (EMT) are critical factors in the onset, advancement, and dissemination of tumours, influencing cellular behaviour and facilitating various stages of tumourigenesis and metastasis [6,7].

EMT is a complex process where epithelial cells undergo a phenotypic shift to a mesenchymal state through reversible changes in gene expression and morphological features. The transition of cells to a more mesenchymal-like phenotype enhances their migratory abilities and is essential for tumour cell invasion of adjacent tissues, subsequent systemic dissemination, and metastatic spread to distant organs [8]. EMT is also crucial for enhancing cancer stem cell (CSC) properties [9,10,11,12], inhibiting apoptosis and senescence, and promoting chemoresistance [13,14]. CSCs are known to contribute significantly to cancer progression, metastasis, and therapeutic resistance due to their self-renewal capacity and multidirectional differentiation ability [15,16,17,18]. During the transition toward the mesenchymal phenotype, epithelial cells undergo a series of significant changes, resulting in the loss of tight cell–cell junctions and polarity. These alterations endow the cells with migratory and invasive properties, thereby facilitating tumour cell dissociation from the primary tumour mass, invasion through the extracellular matrix (ECM) and intravasation into blood vessels [8]. Furthermore, the reverse process from a mesenchymal to an epithelial, known as mesenchymal–epithelial transition (MET), occurs when cancer cells migrating through blood vessels and lymphatic vessels reach other tissues. Here, they are converted back into epithelial cells, re-establishing tight cell–cell junctions and apicobasal polarity at distant sites, thereby promoting metastatic colonisation of distant organs [4] and re-initiation of tumour cell growth [19,20,21].

Therefore, both EMT and MET contribute to cell motility, cancer progression, metastasis, and response to therapy. EMT can be induced by signals derived from tumour cells as well as by various components of the TME. The TME consists of a diverse array of cellular components, including endothelial cells, cancer-associated fibroblasts (CAFs) [22], mesenchymal stromal cells (MSCs) [23], pericytes [24], and immune cells [25,26,27,28,29,30,31], along with non-cellular elements such as ECM proteins [32] and various soluble factors. Immune cells, including lymphocytes [26], B cells [27], natural killer cells (NK) [28], tumour-associated macrophages (TAMs) [25], regulatory T-cells (Tregs) [26], myeloid-derived suppressor cells (MDSCs) [29], tumour-associated neutrophils (TANs) [30], and dendritic cells (DCs) [31], play a crucial role in cancer metastasis and proliferation by directly or indirectly influencing the EMT process. The various components of the TME interact with cancer cells through a complex network of signalling pathways mediated by growth factors [33], cytokines [34], chemokines [35], hormones [33], metabolites, metalloproteases (MMPs) [36], and extracellular vesicles (ECVs) [37], containing non-coding RNA-carrying exosomes and others secreted molecules.

### 1.2. The Role of EMT Transcription Factors in Tumour Cell Motility

**Figure 1:**
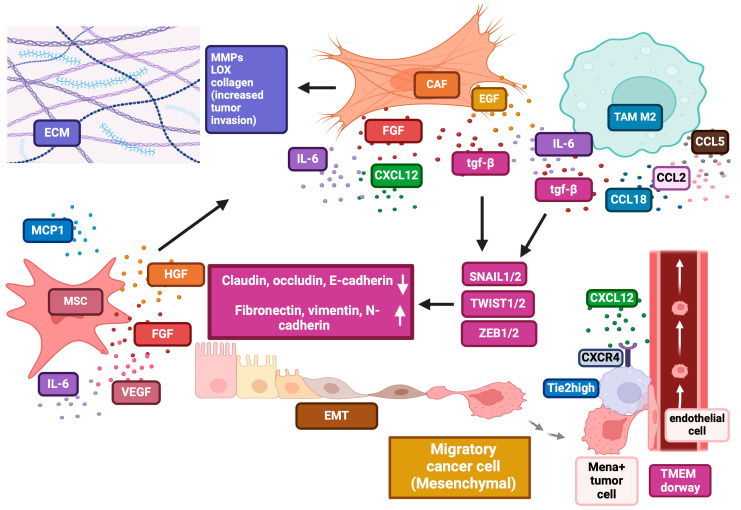
Highlighted are key mechanisms regulating tumour cell motility in the TME, as discussed in the main text. EMT is regulated by TWIST, SNAIL, and ZEB1 transcription factors. CAFs play a crucial role in the activation of EMT by releasing IL-6, TGF-β, CXCL12, among others. Macrophages trigger EMT by releasing TGF-β, CCL2, and IL-6, which result in the upregulation of SNAIL. TMEM doorways consist of stable tri-cellular structures that predominantly form at vascular bifurcations, comprising a Mena expressing tumour cell, a perivascular Tie2High macrophage, and an endothelial cell, promoting tumour cell extravasation and membrane degradation. The pointed arrow indicates activation. This figure was created on Biorender.com (accessed 29 March 2025).

The EMT is orchestrated by several transcription factors (EMT-TFs), including TWIST1/2 (bHLH family), SNAIL1/2 (zinc finger SNAIL family), and ZEB1/2 (zinc finger E-box-binding homeobox family) (Figure 1) [38]. EMT-TFs are activated by diverse TME signals [39], and their upregulation (e.g., ZEB1, SNAIL, and TWIST) is associated with a shift toward a stem-like phenotype in cancer cells [40]. Suppressing these factors may help counteract EMT-related drug resistance [10].

EMT-TFs are modulated by major signalling pathways involving Transforming Growth Factor-β (TGF-β) [41], epidermal growth factor (EGF) [42], insulin-like growth factor-1 [43], hepatocyte growth factor (HGF) [44], WNT/β-catenin [45], and NOTCH [46]. SNAIL1/2 downregulates target genes, such as E-cadherin and MMPS depending on the cellular context [47,48,49], and tight junction proteins (claudins, occludins, ZO-1, and connexins) [48,49,50,51,52]. SNAIL1 also inhibits CRUMBS3, affecting cell polarity [53]. Their overexpression promotes stemness, metastasis [54,55,56,57,58,59,60,61,62,63], and therapy resistance in various cancer models [64] and is associated with poor prognosis and recurrence [59,60,61,62,63,64,65]. SNAIL1 also alters glucose metabolism, shifting cells toward glycolysis [66]. Post-translational regulation is crucial: GSK3-mediated phosphorylation leads to nuclear export and degradation, maintaining epithelial features [67,68], while PAK1-mediated phosphorylation promotes nuclear localisation and EMT [69,70]. TWIST1/2 regulate genes involved in EMT promotion and cancer metastasis [71]. They downregulate E-cadherin and upregulate fibronectin, N-cadherin, and vimentin, facilitating motility and invasion (Figure 1) [72]. TWIST also controls TGFβ2, AKT2, and PDGFR and supports cancer stemness [73,74]. Notably, phosphorylation by MAPK or AKT enhances EMT features [75,76]. ZEB1/2 promote EMT by repressing epithelial and activating mesenchymal genes [77,78] and are regulated by estrogen, TGF-β, and Wnt/β-catenin pathways [79,80]. ZEB1 regulates crucial genes like E-cadherin and MMPs [81] and is modulated by SNAIL1 and TWIST1 [82,83]. The activation of ZEB1/2 ZEB1 represses polarity genes (CDH1, Lgl2, PATJ, Crumbs3), enhancing metastatic potential [83,84]. High ZEB1/2 levels correlate with poor prognosis in various cancers [85,86,87,88,89,90,91].

### 1.3. The Role of Hypoxia

Hypoxia is a pivotal factor in the TME, promoting cell migration, proliferation, apoptosis, and metastasis [92]. It activates hypoxia-inducible factors (HIFs), particularly HIF-1α and HIF-2α, which drive EMT by regulating TFs [93]. HIF-1α can also induce EMT via the TGF-β pathway through SMAD phosphorylation [94].

In breast cancer cells, hypoxia increases uPAR expression, which facilitates SNAIL nuclear translocation, E-cadherin downregulation, vimentin upregulation, and ultimately EMT and migration [95]. Additionally, hypoxia activates NOTCH, NF-κB, and EGFR signalling pathways, further contributing to EMT and tumour progression [96].

HIF-1α enhances metastasis by directly binding hypoxia-responsive elements (HREs) in the TWIST and ZEB1 promoters, as observed in hypopharyngeal, breast, and colorectal cancer cells [97,98]. Overall, hypoxia influences multiple signalling cascades to drive EMT.

### 1.4. The Role of CAFs

CAFs are key TME components that interact with tumour cells and contribute to EMT activation (Figure 1) [99]. Unlike quiescent fibroblasts, CAFs display high metabolic activity and support tumourigenesis through enhanced proliferation, angiogenesis, ECM remodelling, and immune evasion [100].

CAFs encompass several subtypes, including myofibroblast-like CAFs (myCAFs), which remodel and stiffen ECM to aid invasion; inflammatory CAFs (iCAFs), induced by IL-1α or TNF-α, that secrete IL-6, IL-11, LIF, and chemokines to support proliferation, metastasis, and therapy resistance; and antigen-presenting CAFs (apCAFs), expressing MHC-II and modulating T-cell responses to foster immune suppression [101]. Interestingly, apCAFs may also activate CD4^+^ effector T-cells and support anti-tumour immunity in lung cancer [102].

CAFs promote EMT via paracrine release of TGF-β, IL-6, IL-8, IL-32, CXCL12, MMP-2, and MMP-9 [103,104,105,106]. This effect is largely TGF-β-dependent and can be reversed with TGF-β-neutralising Abs or receptor kinase inhibitors, as shown in colon cancer [107]. IL-6 from CAFs also drives EMT via JAK2/STAT3 signalling in lung [108], liver [109], and bladder cancer [110] and amplifies TGF-β signalling transduction [111]. This creates a self-sustaining loop that fuels EMT and drug resistance.

CAFs can also induce EMT through EGF, HGF, and FGF-2 in endometrial cancer and lung metastasis [112] and ovarian cancer by upregulating key EMT markers like SNAIL and MMP3 [113]. CAF-secreted periostin promotes EMT and drug resistance in NSCLC [114]. In pancreatic cancer, EMT induction involves Hedgehog signalling, whose inhibition impairs migration and invasion [115]. Additionally, CAFs remodel ECM through LOX enzymes [116], MMPs [117], and the synthesis of collagen, fibronectin, and proteoglycans, increasing ECM stiffness and promoting invasion [118,119].

In summary, CAF-induced ECM alterations, coupled with tumour–ECM interactions, can drive EMT and modulate cellular behaviours such as adhesion, differentiation, and migration.

### 1.5. The Role of Exosomes

Exosomes are vital TME components that mediate communication with cancer cells (Figure 1) [120,121], serving as crucial signalling molecules to facilitate communication between the TME and cancer cells [122,123]. By transporting proteins, miRNAs, and lncRNAs via endocytosis or systemic routes, exosomes influence gene expression and promote EMT, proliferation, migration, and drug resistance [122,123,124].

CAFs also release exosomes carrying SNAIL, which induce EMT in lung cancer cells [125]. Exosomal RNAs such as miR-21, miR-143, miR-378e, and miR-92a-3p, found in CAF-derived exosomes, promote EMT and drug resistance in different cancer models [126,127,128,129]. In colorectal cancer (CRC), exosomal lncRNA LINC00659 from CAFs upregulates ANXA2, enhancing migration, proliferation, and invasion [130].

### 1.6. Mesenchymal Stromal Cells (MSMCs)

Cancer-associated mesenchymal stromal cells (CA-MSCs) are multipotent progenitors in the TME that support tumour growth through interactions with cancer and stromal cells (Figure 1) [131]. They may also serve as precursors for CAFs.

CA-MSCs secrete factors such as FGF, HGF, MCP-1, IL-6, VEGF, and IL-8, which promote EMT, invasion, metastasis, angiogenesis, ECM remodelling, and immune evasion [132,133,134,135]. They also release TRAIL decoy receptors and immunosuppressive IL-12p40, a soluble IL-2Rα) which hinders anti-tumour immunity [134,135]. MMPs secreted by MSCs degrade the ECM, aiding tumour invasion [136,137].

Human MSCs can activate ADAM metalloproteases in different cancer models, thus increasing the disruption of cell–cell adhesion and enhancing pro-metastatic activity [138,139,140].

### 1.7. The Role of ECM

The ECM is a dynamic and complex structure consisting of various macromolecules, such as collagen, laminin, fibronectin, hyaluronan, and other constituents that influence cellular adhesion, motility, and morphology, thereby facilitating EMT [141]. Collagens are essential structural proteins in the ECM, and their excessive deposition represents one of the most prevalent ECM changes observed in cancer [142]. Collagens can induce EMT through different mechanisms. For example, in NSCLC cell lines, collagens activate TGF-β3 [143], while in pancreatic cancer cells, collagen type I disrupts the E-cadherin adhesion complex [144]. Moreover, collagen type I or type III reduces E-cadherin expression and cell–cell adhesion while promoting proliferation and morphological transformation in pancreatic cancer cells [145].

Fibronectin can significantly influence EMT, migration, invasion, metastatic spread, tumour growth, and angiogenesis by interacting with cellular receptors, such as integrins, as well as ECM components [146]. Breast cancer cells exposed to fibronectin promote EMT by increasing the expression of both N-cadherin and vimentin, thereby enhancing cell migration and invasion [147].

Another ECM constituent that is important in inducing EMT is the linear polysaccharide hyaluronan [148], overproduced in lung cancer cells and associated with pro-tumourigenic effects [149]. The binding of hyaluronan to the CD44 glycoprotein on the surface of breast cancer cells induces LOX transcription, leading to TWIST1-induced EMT, thereby enhancing metastatic dissemination [150].

### 1.8. TAM, Tumour-Associated Macrophages

TAMs primarily originate from monocytic progenitor cells [151], although a significant fraction in some tumours derive from tissue-resident macrophages [152]. Cancer cells secrete chemokines like CCL2/MCP-1 [153], CCL5 [154], and CCL8/MCP-2 [155], which recruit TAMs into the TME. TAMs regulate tumour progression by influencing proliferation, EMT, metastasis, ECM remodelling, angiogenesis, immune modulation, genetic instability, and therapy resistance [156]. Through cross-talk in the TME, tumour cells reprogram TAMs to enhance growth factor production (Figure 1), promoting EMT and tumour progression. Macrophages trigger EMT by releasing TGF-β [157], CCL2 [158], and IL-6 [159], which increase SNAIL levels (Figure 1). IL-6, in turn, downregulates tumour-suppressive miR-34a and promotes EMT, tumour growth, and dissemination [160,161].

CCL2 and IL-6 secreted by TAMS attract more macrophages [162] and foster M2 polarisation [163], perpetuating the EMT feedback loop. In breast cancer, TAMs induce EMT by secreting TGF-β1, CCL18, CCL2, and IL-6 [164,165]. Exosomes facilitate the feedback loop between macrophages and tumour cells supporting EMT and M2 polarisation [166,167].

CCL18, primarily secreted by TAMs, activates NF-κB through PITPNM3, enhancing EMT gene expression and promoting cancer cell migration and metastasis [168,169]. CCL18 also stimulates ELMO1 and PI3K/AKT, driving tumour cell motility and reinforcing EMT [170]. TAMs are linked to increased vimentin and decreased E-cadherin, enhancing migration and invasion in several cancers [171], with TAM-derived IL-6 [172] and EGF [173] contributing to this process.

TAMs remodel the ECM, increasing stiffness and deposition in both cancerous and normal tissues [174,175]. Matrix degradation via MMPs, driven by STAT3, ERK, and NF-κB, is crucial for cancer progression [176,177,178,179]. TAMs release TGF-β, IL-1β, and IL-6, which increase MMP-9 in tumour cells [176,177,178]. In gastric cancer, IL-1β upregulate MMP-9, facilitating invasion [180]. MMP-11 from breast cancer TAMs is a poor prognostic marker, which promotes migration via MMP-9 expression [181]. MMP-9 also activates PI3K/AKT, driving EMT and enhancing invasion [182,183].

TAMs support tumour cell migration towards blood vessels, facilitating intravasation and systemic dissemination. Monocytes differentiate into motile CXCR4-expressing TAMs, which are recruited by CXCL12-expressing fibroblasts to perivascular niches. TAMs form TMEM doorways with tumour cells and endothelial cells, stimulating VEGFA secretion, which disrupts endothelial junctions and increases vascular permeability (Figure 1) [184,185].

Invadopodia formation is crucial for intravasation [186]. TAM–tumour interactions drive invadopodia formation, leading to matrix degradation and intravasation [186,187]. TAMs enhance their intra-tumour persistence by promoting invadopodia assembly via actin-regulatory proteins [187,188,189]. TAMs also support invadopodia maturation by inducing the Mena^INV variant, which stabilises and prologues the degradative activity of invadopodia [190]. Moreover, TAMs release chemotactic signals like CSF1 and EGF [191,192,193,194,195], while HGF from endothelial cells acts as a chemoattractant for macrophage–tumour co-migration [196]. In conclusion, TAMs collaborate with tumour cells to enhance migration towards blood vessels and facilitate invadopodia formation, ultimately promoting tumour dissemination.

### 1.9. Metabolism and Tumour Migration

Cell migration and invasion are energy-dependent processes reliant on actin cytoskeleton dynamics and ATP consumption [197]. Cancer cells adapt by directing metabolic proteins to high-energy areas, with glycolytic and mitochondrial pathways playing key roles. Glycolysis, although less energy-efficient, provides rapid ATP for cytoskeletal activity, while mitochondrial respiration ensures sustained energy. Cancer cells often favour glycolysis, a phenomenon known as the “Warburg effect”, which increases glucose uptake to support invasive growth [198]. Under hypoxia, HIF signalling further drives this glycolytic shift, creating an acidic microenvironment that enhances invasion by suppressing immune responses and activating proteases [199]. These metabolic shifts also promote the use of alternative energy sources like glutamine, amino acids, lipids, and dietary sugars, which increase motility and metastatic potential. For instance, glutamine metabolism is critical for invasion and linked to higher colorectal cancer metastasis risk [200]. Non-essential amino acids, such as asparagine, promote migration and metastasis by influencing processes like EMT in various cancers [201].

Lipid metabolism is also pivotal in the TME, supporting cancer cell growth and survival through metabolic interactions with CAFs and adipocytes. Hypoxia promotes lipid droplet formation in cancer cells, which serve as energy reservoirs [202]. Targeting lipid metabolic pathways, including fatty acid oxidation (FAO) and lipid uptake, shows promise as a therapeutic strategy. Pre-clinical studies suggest that inhibiting lipogenesis enzymes and fatty acid uptake can reduce tumour growth [203].

In conclusion, targeting metabolic pathways in cancer cells and the TME represents a promising strategy to hinder tumour growth and improve treatment outcomes.

## 2. Regulation of Intra-Tumour T-Cell Motility: Dynamics, Mechanisms, and Implications

### 2.1. Groundwork of Intra-Tumour T-Cell Motility: Concepts and Challenges

Immune checkpoint blockade (ICB), particularly targeting CTLA-4 or PD-1/PD-L1, has significantly advanced cancer treatment, offering clinical benefits across various cancers, including melanoma, NSCLC, and renal cell carcinoma [204,205,206,207]. However, resistance remains an issue, requiring deeper investigations into the underlying mechanisms and the identification of biomarkers predictive of ICB response [208]. Patients who respond to ICB often exhibit a “hot” (immune-inflamed) TME, characterised by immune cell infiltration and activation. In contrast, non-responders typically display a “cold” (immune desert) phenotype, lacking immune infiltration [209]. A more complex “excluded” phenotype, where immune cells are at the tumour periphery but fail to infiltrate the core, has also been identified [209]. Additionally, an “immunosuppressive tumour” subtype, characterised by high immune cell infiltration but dominated by immunosuppressive cells, is associated with poor prognosis [210]. These findings highlight the need for patient-tailored immunotherapeutic strategies based on the tumour’s immune landscape [206,210].

To convert a cold TME into a hot, immune-active environment, strategies must focus on enhancing immune cell infiltration, overcoming immunosuppressive barriers, and stimulating an effective anti-tumour immune response. Understanding tumour-intrinsic factors, the cross-talk between tumour and immune cells and the influence of external factors is critical for predicting responses and tailoring treatments. Combination therapies are being developed to reprogram the TME, promoting immune cell infiltration and transforming cold or excluded tumours into a hot state [211,212].

Enhanced T-cell motility is essential for effective tumour infiltration, allowing immune cells to locate and attack cancer cells. Increased motility improves T-cell interactions with antigen-presenting cells (APCs), boosting anti-tumour responses. In the context of immune checkpoint inhibition, enhanced motility helps T-cells overcome barriers like stromal density, hypoxia, and immunosuppressive signals, ultimately leading to better tumour control and reduced resistance to immunotherapy [213,214]. Chemokines play a key role in directing T-cell subtypes, including CD4^+^, CD8^+^, and Tregs, to different immune niches [215,216].

The TME is a dynamic system where immune cells interact with each other, tumour cells, and stromal components, influencing cancer progression. Immune cell subsets within the TME modulate migration through cell–cell interactions, soluble factors, and ECM modifications, all of which impact the efficacy of IC inhibitors (ICIs) and other therapies [217,218].

Pre-existing infiltration of CD8^+^ T-cells within tumours is a key factor in the efficacy of IC therapy [219,220]. Intra-tumour T-cell motility is influenced by both intrinsic factors and external cues such as matrix proteins, chemokines, and antigen density [213]. Understanding these mechanisms is essential for optimising cancer immunotherapy and developing more effective strategies.

### 2.2. Chemokines and Their Role in the Regulation of Intra-Tumour T-Cell Migration

Chemokines are key signalling molecules that regulate immune cell migration through chemotaxis, influencing both immune surveillance and anti-tumour responses. These small proteins act as chemotactic cytokines, activating G protein-coupled receptors to guide immune cells within tissues [221]. Based on conserved cysteine residues, chemokines are classified into four subfamilies: CX3C, CXC, CC, and C [221]. Within the TME, chemokines secreted by host and tumour cells can recruit immune cells, mediating either pro-tumour or anti-tumour responses. CXCR3, a CXC chemokine receptor, plays a pivotal role in regulating tumourigenesis, angiogenesis, and immune cell infiltration. This duality is influenced by different CXCR3 variants (CXCR3-A, CXCR3-B, CXCR3-alt), which differ in their ligand-binding properties, signalling pathways, and cellular responses [222].

**Figure 2:**
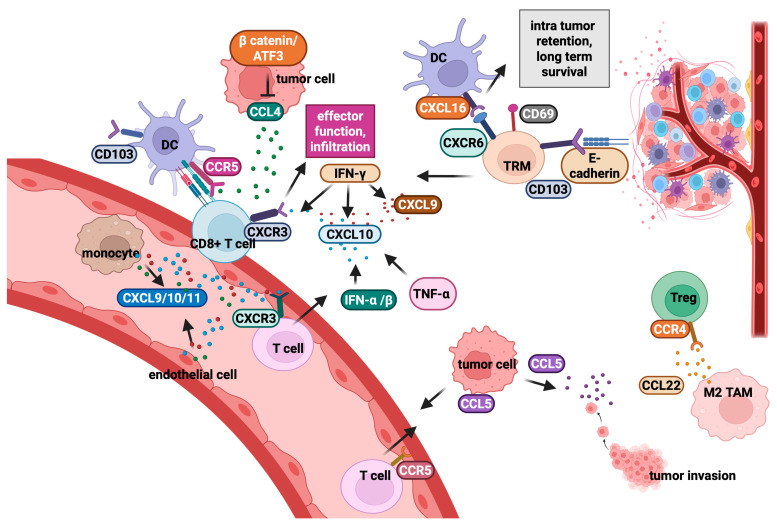
Highlighted are key mechanisms regulating intra-tumoural T-cell infiltration, as discussed in the main text. IFN-γ is a major driver of the CXCR3–CXCL9/10/11 axis, promoting T-cell recruitment. CCL4 recruits CD103^+^ DCs via CCR5, facilitating CD8^+^ T-cell accumulation. β-catenin signalling suppresses CCL4 transcription through ATF3, impairing CD103^+^ DC recruitment and subsequent T-cell infiltration. CCL5 is associated with cancer cell migration, but it also enhances CD8^+^ T-cell recruitment into the TME. CCL22 primarily binds CCR4, which is expressed on Tregs, promoting their accumulation. CD103 binds to E-cadherin, facilitating the retention of tissue resident T_RM_ within the TME. The CXCR6/CXCL16 axis plays a critical role in intra-tumoural T-cell retention. T_RM_ cells enhance the recruitment of circulating T lymphocytes by producing IFN-γ, which upregulates chemokine release. The pointed arrow indicates activation; the flat-tipped arrow indicates inhibition. This figure was created on Biorender.com (accessed 18 April 2025).

Effector cells like CD8^+^ T-cells and NK cells rely on CXCR3 to infiltrate tumours [223]. CXCR3 ligands, such as CXCL9, CXCL10, and CXCL11, are mainly produced by monocytes, endothelial cells, and tumour cells [224,225] and are crucial for regulating anti-tumour immunity. Elevated expression of these ligands correlates with enhanced immune cell infiltration and better therapeutic outcomes [215,221,224,225]. IFN-γ is a major inducer of the CXCR3–CXCL9/10/11 axis (Figure 2). While CXCL9 is induced by IFN-γ but not by type-1 interferons (IFN-α/β), CXCL10 is inducible by both IFN-γ and type-1 IFNs as well as tumour necrosis factor alpha (TNF-α) (Figure 2) [215,226,227]. In cutaneous T-cell lymphoma, single-cell RNA sequencing has shown that CXCR3 ligands, particularly CXCL9 and CXCL11, are primarily derived from macrophages, with higher expression linked to increased CD8^+^ T-cell infiltration [215]. Notably, CXCR3 expression can serve as a biomarker for the effectiveness of anti-PD-1 mAbs. Blocking CXCR3 impairs the efficacy of PD-1 blockade, whereas enhancing its expression promotes T-cell infiltration and tumour suppression [228].

The expression of CXCR3-A on tumour cells is associated with greater proliferation and spreading, whereas CXCR3-B limits these processes [222]. A comprehensive understanding of CXCR3 variants and their distinct signalling pathways is essential for evaluating their potential as anti-tumour targets and for developing variant-specific drugs for combination therapies. CXCR3 expression on NK cells plays a dual role in the TME: it can enhance NK cell recruitment and promote a highly cytotoxic phenotype [221,223], but persistent CXCR3 signalling in an immunosuppressive TME may lead to NK cell dysfunction and impaired tumour clearance, contributing to immune evasion [229]. However, genetic deletion of CXCR3 or in vivo administration of anti-CXCR3 mAbs has been shown to enhance bone marrow NK cell infiltration, improving anti-myeloma efficacy [230] and promoting tumour rejection in melanoma models [231,232].

The recruitment of CD103^+^ DCs by CCL4 occurs via CCR5 (Figure 2), an essential step for successful CD8^+^ T-cell accumulation through the CXCL9/10 signalling axis [233]. In “cold” tumours, β-catenin signalling suppresses CCL4 transcription via activation of the transcription factor ATF3 (Figure 2), thus impairing CD103^+^ DC recruitment and the associated T-cell infiltration [234]. Immunohistochemical analyses and database studies have shown that higher CCL4 levels correlate with improved survival in primary melanoma patients [235], while T-cell single-cell RNA sequencing in metastatic melanoma patients suggests its association with durable responses to ICB [236]. In NSCLC, aberrantly high CCL4 expression has been linked to immune infiltration [237], although it correlates with reduced CD8^+^ T-cell survival and elevated PD-1/PD-L1 expression. Furthermore, intra-tumour delivery of CCL4 has been reported to enhance CD103^+^ DC and CD8^+^ T-cell recruitment in melanoma and breast cancer models [238].

CCL5, also known as Regulated upon Activation Normal T-cell Expressed and Secreted (RANTES), binds primarily to CCR5 but can also interact with CCR1, CCR3, and CCR4 and is induced by IFN-γ, IL-1, and TNF-α [239]. Produced by tumour cells, lymphocytes, and myeloid cells, CCL5 plays a dual role in immune activation and tumour immune evasion [240]. The CCL5/CCR5 axis promotes tumour growth, ECM degradation, and cancer cell migration (Figure 2), while CCR5^+^ cancer cells exhibit stem-like properties, contributing to therapy resistance. Additionally, CCL5 induces metabolic reprogramming, enhances angiogenesis, and recruits normal cells to support tumour development [241]. CCL5 also shapes an immunosuppressive TME by polarising monocytes and myeloid cells into tumour-promoting phenotypes, leading to effector T-cell exhaustion [240]. Nonetheless, CCL5 is part of the T-cell-inflamed gene signature, and higher levels correlate with T-cell infiltration in melanoma metastases and CD8^+^ T-cell recruitment to the TME (Figure 2) [242]. Recent studies suggest that CCL5 levels are positively associated with the infiltration of CD8^+^ T cells, B cells, DCs, and macrophages and negatively associated with myeloid progenitor cell infiltration [243,244,245]. These findings highlight the dual role of the CCL5/CCR5 axis, recruiting both effector T-cells and immunosuppressive compartments depending on local cues [246].

CCL22, produced by both tumour and immune cells [247,248], primarily binds to CCR4, which is expressed on Tregs (Figure 2), Th2-polarised T-cells, and specific DC subsets. CCL22 facilitates tumour progression and immune evasion by recruiting Tregs, thereby establishing an immunosuppressive TME. Elevated CCL22 levels have been observed in melanoma and other cancers, promoting the accumulation of Foxp3^+^ Tregs, often in collaboration with CCL17 [249,250]. IL-1α, secreted by cancer cells, has been identified as a driver of CCL22 production, further amplifying Treg recruitment and suppressing anti-tumour immunity [251]. These findings emphasise the complex, context-dependent role of chemokine signalling in cancer, with CCL4 enhancing T-cell infiltration, CCL5 mediating both immune activation and suppression [252], and CCL22 driving immune evasion through Treg recruitment.

Tissue resident memory T (T_RM_) cells, characterised by markers such as CD103, CD49a, and CD69, are essential for immunosurveillance in epithelial tissues. CD103 binds to E-cadherin (Figure 2), facilitating intra-tumour T_RM_ retention. T_RM_ cells contribute significantly to tumour immunology, enhancing localised immune responses and improving immunotherapy efficacy [253,254]. The CXCR6/CXCL16 axis is crucial for T_RM_ cell retention in tumours (Figure 2) [255]. CXCR6 enhances the interaction between CXCR6^+^ cytotoxic T-cells and CXCL16^+^ DCs, supporting T-cell retention and function within the TME [256]. CXCR6-deficient T-cells or those treated with CXCL16-blocking Abs show impaired retention and reduced local anti-tumour activity. Additionally, CXCR6-deficient mice exhibit increased tumour metastasis in liver and lung models, highlighting the importance of this axis in limiting metastatic spread [257]. Interestingly, blocking CXCL16 in metastatic lung models of breast cancer redirects T-cells to distant lung tissues, reducing the metastatic tumour burden [258].

Increased infiltration of T_RM_-like TILs correlates with improved survival in lung cancer patients, emphasising their role in anti-tumour immunity. However, many T_RM_-like TILs lack tumour specificity, and their exact function in orchestrating anti-tumour responses in lung cancer remains unclear [259]. Preclinical studies suggest that alveolar CD8^+^ T_RM_ cells migrate from the parenchyma to the airways, replacing short-lived CD8^+^ T_RM_ cells while downregulating CXCR6 and CD11a expression [260]. TRM-like TIL infiltration also correlates with higher overall TIL numbers across various NSCLC histological subtypes, particularly in smokers, and is associated with improved outcomes [261]. TRM cells enhance the recruitment of circulating T lymphocytes by producing IFN-γ (Figure 2), which upregulates chemokine release and vascular adhesion molecule expression [262,263,264,265]. CD8^+^ CD103^+^ CD39^+^ PD-1^+^ CD28^-^ TRM cells, known for their higher anti-tumour cytokine production, may facilitate the recruitment of less-exhausted peripheral T-cells to the NSCLC tumour site, potentially improving their response to ICIs [266]. Marceaux et al. have reviewed the pivotal role of the CXCR6/CXCL16 axis in lung T_RM_ cell motility [267].

**Figure 3:**
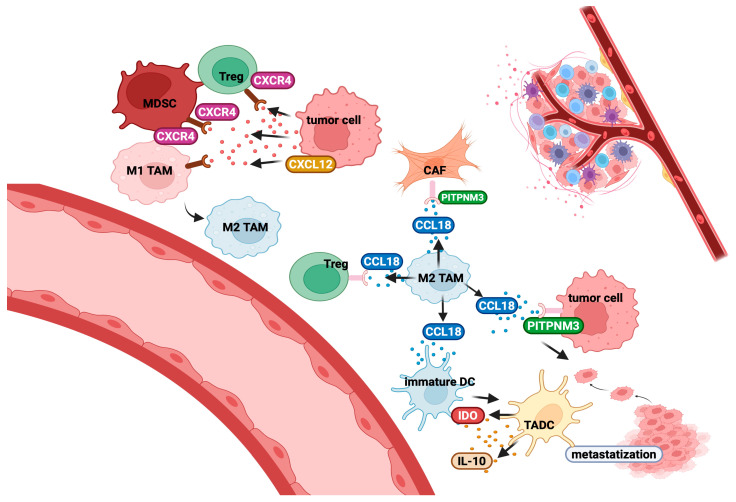
Highlighted are key mechanisms regulating intra-tumoural T-cell infiltration, as discussed in the main text. CCL18 produced by M2 TAMs facilitates the recruitment of Treg, CAFs, and immature DCs via its receptor PITPNM3 to the tumour niche. Immature DCs then differentiate into tumour-associated dendritic cells (TADCs), which promote tumour progression by secreting IL-10 and IDO. CXCL12 secreted by tumour cells facilitates the recruitment of Tregs and MDSCs while inducing immunosuppressive M2 TAM switch. The pointed arrow indicates activation. This figure was created on Biorender.com (accessed 18 April 2025).

CCL18 is a critical chemokine in the TME, primarily secreted by TAMs (Figure 3) but also by CAFs and tumour cells [268,269]. It plays a significant role in tumour progression by promoting cancer cell migration, EMT, immune suppression, and interactions between stromal and immune cells. In various cancers, including colon and renal carcinoma, CCL18 expression is enhanced by the β-catenin pathway [270], highlighting its role in the tumour adaptive response. CCL18 exerts pro-tumourigenic effects through two main mechanisms. First, it directly enhances cancer cell motility and metastasis, primarily via the PITPNM3 receptor [271], involved in pathways driving tumour proliferation, EMT, and invasion [272,273]. Second, CCL18 modulates the TME by recruiting and sustaining TAMs and CAFs, fostering an immunosuppressive niche. CCL18 enhances TAM–CAF interactions and facilitates the recruitment and differentiation of Tregs via its receptor PITPNM3 (Figure 3) [274,275]. Tregs, in turn, reinforce TAM polarisation towards a pro-tumourigenic M2 phenotype, promoting further CCL18 production [276]. Additionally, CCL18 recruits naïve T-cells, which differentiate into Tregs upon exposure to TAMs [277] and immunosuppressive IL-10 [276].

CCL18 also influences DC fate by attracting immature DCs to the tumour niche, where they differentiate into tumour-associated DCs (TADCs) [273,278], which produce immunosuppressive IL-10 and indoleamine 2,3-dioxygenase (IDO) (Figure 3) [279], further expanding Tregs.

Within the TME, CXCL12 and its receptor CXCR4 promote tumour angiogenesis, sustain tumour cell survival and proliferation, and orchestrate immunosuppressive responses [280]. CXCL12 is secreted by tumour cells (Figure 3) across multiple cancer types, including advanced cervical cancer, malignant pleural mesothelioma, ovarian cancer, and renal cell carcinoma, facilitating the recruitment of TAMs and Tregs [281]. The CXCL12/CXCR4 axis also supports the recruitment of monocytes and induce their shift towards an immunosuppressive M2 phenotype (Figure 3) [281,282].

Additionally, MDSCs are attracted via CXCL12-CXCR4 signalling (Figure 3), further suppressing anti-tumour immune responses and promoting tumour growth [283]. B cells are similarly affected by CXCL12/CXCR4 interactions. Indeed, CXCL12 recruits regulatory B cells (Bregs) to the tumour site, where they suppress T-cell activity [284].

CXCL12 binding to CXCR4 also activates key signalling pathways, triggering calcium mobilisation and actin polymerisation, that are essential processes for T-cell motility [281]. Importantly, CXCR4 heteromerises with CCR7, enhancing CCR7 ligand binding and promoting T-cell retention in lymphoid tissues [285].

CAFs also play a role in creating a chemical barrier by secreting CXCL12, attracting CXCR4-expressing T-cells. Through the CXCL12–CXCR4 axis, CAFs misdirect CTLs to the stromal area, preventing their effective targeting and elimination of cancer cells [210].

CXCL13, another critical chemokine, binds to CXCR5 and facilitates immune cell trafficking [286]. TGF-β has been identified as an inducer of CXCL13 production in T-cells [287], and follicular helper T-cells (Tfh) have been identified as the primary source of CXCL13 secretion in secondary lymphoid organs (Figure 3).

A critical function of CXCL13 is its role in the development and maintenance of lymphoid follicles, where it recruits B cells and T-cells to specific regions within lymphoid-like structures [286]. In the TME, CXCL13 is a hallmark of tertiary lymphoid structures (TLSs) (Figure 3) [288], which facilitate antigen presentation and support effective anti-tumour T-cell responses [289]. However, in many cancer types, the CXCL13/CXCR5 axis also promotes the recruitment of immunosuppressive cells to the tumour site.

**Figure 4:**
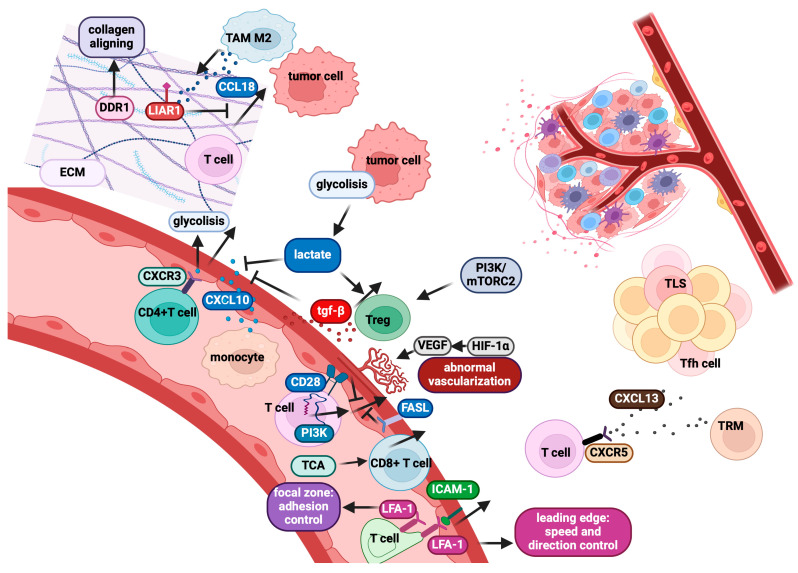
Highlighted are key mechanisms regulating intra-tumoural T-cell infiltration, as discussed in the main text. T-cell migration and infiltration into the TME are influenced by a complex interplay of chemokines, adhesion molecules, metabolic pathways, and ECM remodelling. CXCL13, mainly produced by Tfh in TLS and T_RM_ cells, binds CXCR5, facilitating immune cell trafficking. LFA-1 forms specialised structures called podosomes, which interact with ICAM-1-enriched endothelial invaginations, enabling transcellular migration. Within migrating T-cells, LFA-1 in the focal zone controls adhesion, while LFA-1 at the leading edge regulates speed and direction. CD28 signalling promotes T-cell migration from lymphoid tissues to sites of inflammation, requiring PI3K-dependent motility and trafficking regulation. The ECM significantly influences T-cell migration to tumours. Abnormal ECM remodelling in the TME generates a dense, stiff matrix, acting as a physical barrier that restricts T-cell infiltration. The collagen receptor DDR1 contributes to immune evasion by aligning collagen fibres, further limiting T-cell movement. CCL18 enhances collagen receptor LAIR1 expression. HIF-1α upregulation promotes pro-angiogenic VEGF-A, leading to excessive neovascularisation and the formation of structurally abnormal blood vessels. Additionally, endothelial FASL expression correlates with CD8^+^ T-cell exclusion from tumours. The glycolytic pathway is required for CXCL10-induced migration of activated T-cells. However, lactate accumulation impairs CD4^+^ T-cell motility by disrupting glycolysis, particularly upon CXCR3-CXCL10 engagement, whereas CD8^+^ T-cell motility primarily relies on TCA cycle metabolism. Treg migration depends on a PI3K/mTORC2 pathway, activated through integrins and chemokine receptors. The pointed arrow indicates activation; the flat-tipped arrow indicates inhibition. This figure was created on Biorender.com (accessed 29 March 2025).

Preclinical models have demonstrated that the CXCL13/CXCR5 axis is crucial for promoting intra-tumour T-cell migration (Figure 4), and accordingly, tumours with high CXCL13 expression exhibit an increased infiltration of activated CD8^+^ CXCR5^+^ T-cells. These findings underscore the CXCL13 therapeutic potential as a chemoattractant in enhancing immunotherapy responses [290]. Interestingly, recent preclinical studies have reported that CXCL13^+^ T-cells within TLSs predict favourable prognosis, whereas their presence outside TLSs correlates with poorer outcomes [291]. Nevertheless, further studies are needed to validate the clinical relevance of CXCL13 as a predictive biomarker for immunotherapy efficacy.

### 2.3. Modulation of Adhesion Processes Excludes Anti-Tumour T-Cells: LFA-1 as a Critical Mediator

Lymphocyte function-associated antigen-1 (LFA-1) plays a critical role in the formation of the immunological synapse (IS) between T-cells and APCs [292], initiating cell adhesion by interacting with intracellular adhesion molecule 1 (ICAM-1). During IS formation, T-cells receive signals from chemokines, such as CCL19 and CCL2 [293].

T-cell migration is a critical process for immune surveillance, allowing T-cells to move across various tissues to protect the host and maintain homeostasis. This process, known as extravasation, involves T-cell motility from circulation into inflamed tissues or lymph nodes, which is essential for immune function and maturation [294].

LFA-1 can exist in different affinity states that play distinct roles in migration [295,296]. In the high-affinity state, activated by chemokine signalling and TCR engagement, LFA-1 mediates firm adhesion. In contrast, the intermediate-affinity state, induced by selectin engagement, allows for reversible interactions with ligands like ICAM-1. This reversible binding slows T-cell rolling, promoting chemokine receptor engagement and migration. T-cell crawling and trans-endothelial migration rely on chemokine-triggered high-affinity LFA-1 [297], which reorganises the cytoskeleton, promoting adhesion and spreading. These interactions guide T-cell migration through endothelial cells into tissues. LFA-1 forms podosomes that interact with ICAM-1-rich invaginations in the endothelium, aiding transcellular migration (Figure 4). LFA-1 also acts as a mechanical sensor, with shear forces increasing filopodia density, helping T-cells locate extravasation sites [296]. Additionally, LFA-1 activation is influenced by cytoskeletal dynamics, with actin polymerisation and myosin contraction inducing cell polarisation. The leading-edge forms protrusions interacting with the extracellular environment, where LFA-1 regulates migration speed and direction, while LFA-1 at the focal zone controls adhesion (Figure 4) [296].

### 2.4. Role of CD28 and CTLA-4: Key Regulators of T-Cell Activation and Migration

During T-cell activation, CD28 binds to its ligands, CD80 and CD86, on APCs to sustain immune responses [206]. Beyond its role in immune modulation, CD28 is essential for cellular reorganisation, cytokine and chemokine production, and key biochemical processes such as phosphorylation, transcription, and metabolism, all of which support T-cell growth, specialisation, and T-cell infiltration [298,299].

Furthermore, CD28 signalling enhances IS formation by strengthening the adhesion between T-cells and APCs (including tumour cells). This interaction is crucial for effective antigen recognition and T-cell activation during the early immune response. CD28 signalling involves key molecules such as the lipid kinase PI3K and LCK [206,300], which recruits protein kinase C [301]. These signalling pathways influence the organisation of the cytoskeleton and promote T-cell localisation to target tissues following antigenic priming [302]. Notably, CD28 signalling drives T-cell migration from lymphoid tissues to sites of inflammation. The CD28-mediated regulation of T-cell motility and trafficking needs an intact CD28 signalling and, in particular, PI3K activity (Figure 4) [303]. While the downstream pathways linking CD28 to adhesion and migration remain incompletely understood, a loss of CD28 binding to PI3K alters tissue localisation [303].

Naïve T-cells explore short distances for antigens, while primed T-cells migrate longer distances to infection sites [304]. Co-receptors like CD28 and CTLA-4 regulate migration pathways. CTLA-4 deficiency causes extensive organ infiltration, suggesting its role in T-cell motility regulation. Evidence from mice with tailless CTLA-4 mutants shows altered migration patterns [305]. Accordingly, depletion of CTLA-4 in T-cell subpopulations reveals its role in preventing excessive infiltration into non-lymphoid tissues, with Tregs preventing activation and conventional T-cells preventing excessive migration [306].

At variance, CTLA-4 has also been reported to impact T-cell movement by activating LFA-1 adhesion through integrin clustering and PI3K binding [307,308]. This suggests CTLA-4 generates both positive and negative intracellular signals, as seen in NGF signalling [309]. CTLA-4 prevents stable conjugates with APCs, enhancing T-cell motility while limiting DC binding in the “reverse-stop signal model” [310]. Without CTLA-4, antigen-specific T-cells migrate despite antigen presence, reducing contact with DCs and raising activation thresholds, but this effect is absent in Tregs or anergic T-cells [311,312]. CTLA-4 upregulates chemokine receptors like CCR5 and CCR7, enhancing sensitivity to CCL4, CXCL12, and CCL19 [309], and modulates chemotaxis by controlling receptor phosphorylation and desensitisation [309]. Anti-CTLA-4 Abs enhance T-cell motility and reduce T-cell contact with APCs, weakening TCR signalling [309]. CTLA-4 and PD-1 influence T-cell migration and interactions with APCs in a context-dependent manner. Tumours, especially in immune-privileged sites like the CNS, upregulate CTLA-4 at barriers to limit T-cell infiltration [313,314]. Anti-CTLA-4 blockade enables T-cell tumour infiltration, enhancing immune responses, as seen in murine breast cancer models. Interestingly, NKG2D expression in TILs counteracts this motility by promoting arrest [315].

### 2.5. The Role of Stromal and ECM Components in Intra-Tumour T-Cell Motility

Efficient T-cell migration to the tumour site is crucial for the prognosis and success of cancer immunotherapy. However, ECM abnormalities in structure and stiffness within the TME often hinder T-cell infiltration [316,317]. The TME is a dynamic ecosystem comprising tumour, immune, and stromal cells, where ECM provides structural support and regulates tumour progression, immune evasion, and T-cell migration [318].

Abnormal ECM remodelling creates a dense, stiff matrix that impedes T-cell movement (Figure 4). Excessive collagen deposition increases matrix stiffness, limiting T-cell infiltration [319]. Key ECM components, such as collagen, fibronectin, and glycosaminoglycans, influence cell adhesion and motility. T-cell surface receptors, including integrins and CD44, interact with these ECM molecules to govern T-cell migration [320]. Aberrant ECM composition can disrupt these processes, as observed with DDR1, which promotes immune evasion by aligning collagen fibres and limiting T-cell infiltration (Figure 4). Increased ECM stiffness and altered composition also impact chemokine gradients guiding T-cell migration. CCL18 contributes to resistance to anti-PD-1/PD-L1 therapy in lung tumours by increasing collagen receptor LAIR1 levels (Figure 4), which suppress T-cell motility and activation, reduce proliferation, and skew the T-cell population toward a higher CD4^+^/CD8^+^ ratio, indicating fewer cytotoxic cells. Transcriptomic data show downregulation of cytotoxic genes and upregulation of suppressive markers in T-cells exposed to dense collagen, mirroring reduced CD8^+^ T-cell infiltration in collagen-rich tumours [321]. Conversely, inflammatory signals like TNF-α, IFN-γ, and TGF-β in ECM-dense tissues can induce protease secretion to loosen the matrix and facilitate T-cell motility [322,323]. The ECM also actively contributes to immune suppression by interacting with immunosuppressive cells. For example, collagen I within the ECM can affect T-lymphocyte composition and activity [324]. Moreover, TAMs and CAFs interact with the ECM to recruit and retain immunosuppressive cells [325]. In conclusion, the ECM, along with stromal cells like TAMs and CAFs, creates a microenvironment that not only hinders immune cell infiltration but also recruits immunosuppressive cells, effectively shielding tumours from immune surveillance.

### 2.6. Tumour Vasculature Can Limit CD8^+^ T-Cell Infiltration into Tumours

Tumour vasculature plays a critical role as a barrier to CD8^+^ T-cell infiltration, limiting immunotherapy efficacy [326,327,328]. Effective T-cell entry into tumours is crucial for therapeutic success, but even when antigen-specific T-cells are in the bloodstream, tumour infiltration is often inadequate [329]. Several tumour-derived factors, including VEGF, CD73, and endothelin B receptor (ETBR), suppress vascular adhesion molecules, restricting T-cell entry. However, effector CD8^+^ T-cells can enhance their infiltration through positive feedback loops. For example, intratumoral IFN-γ secretion upregulates chemokines like CXCL10, attracting additional T-cells, and anti-PD-1 therapy amplifies this process by boosting IFN-γ production and chemokine expression [330]. T-cells extravasate through the vasculature in a multistep process to enter peripheral tissues, including tumours [331]. The homing receptors and ligands required for T-cell entry are determined by T-cell activation, local inflammation, and the anatomy of the target tissue [332,333]. Chronic inflammation can induce lymphoid neogenesis in solid tumours, leading to the formation of TLS with HEV-like Peripheral Node Addressin^+^ (PNAd^+^) vasculature [334,335]. Tumour vasculature disorganised structure is a major barrier to T-cell infiltration. Rapid tumour expansion outperforms angiogenesis, resulting in tissue hypoxia [336]. HIF-1α upregulation in tumour and endothelial cells promotes pro-angiogenic genes like VEGF-A, leading to excessive neovascularisation and structurally abnormal blood vessels (Figure 4) [337]. These defects create an inhospitable environment for T-cell infiltration due to irregular blood flow, compromised endothelial junctions, and altered endothelial cell cytoskeletons that impairs transmigration.

Beyond structural issues, tumour-associated endothelial cells often exhibit impaired responses to inflammatory signals. VEGF-A and FGF-2 suppress TNF-α-induced adhesion molecule expression on endothelial cells, further restricting T-cell entry [338].

FAS ligand (FASL/CD95L), a regulator of T-cell homeostasis through apoptosis [298], is also expressed on tumour vasculature in humans and mice [339]. Endothelial FASL expression correlates with the exclusion of intra-tumoral CD8^+^ T-cells (Figure 4), while Tregs remain unaffected. In mouse models, endothelial FASL limits T-cell infiltration, preferentially eliminating tumour-reactive CD8^+^ effector T-cells while sparing Tregs, skewing the CD8^+^/Foxp3^+^ T-cell ratio, and promoting immune tolerance and tumour progression [340].

### 2.7. Metabolic Control of CD8^+^ T-Cell Motility and Implications for Immunotherapy

The TME imposes structural and metabolic barriers that hinder CD8^+^ T-cell infiltration and function. TAMs and ECM fibres sequester CD8^+^ T-cells within stromal regions, preventing direct interaction with cancer cells [341]. These physical barriers also restrict CD8^+^ CAR T-cell infiltration, posing a challenge for immunotherapies targeting solid tumours. Beyond extracellular constraints, cell-intrinsic metabolic limitations also affect T-cell motility [342]. T-cell migration is energy-consuming, requiring ATP and GTP for cytoskeletal remodelling. While oxidative phosphorylation (OXPHOS) and glycolysis are implicated, the metabolic pathways involved are incompletely understood. Studies suggest that glucose and glutamine deprivation negatively impacts T-cell migration [343], with aerobic glycolysis often identified as the primary ATP source for migration [344]. Glycolysis occurs in the cytoplasm, providing localised ATP production for actin polymerisation [345]. Chemokine signalling can modulate metabolic pathways; for example, CCL5 stimulates mTORC1-dependent glucose uptake and activates AMPK [343]. Glycolysis is also required for CXCL10-induced T-cell migration (Figure 4) [346], and Treg migration depends on the PI3K/mTORC2 pathway activated by integrins and chemokine receptors (Figure 4) [347].

Cytoskeletal proteins interact with glycolytic enzymes, linking glycolysis to cytoskeletal dynamics [348]. PFK binding to actin enhances glycolysis, amplified by insulin signalling [349]. Metabolic byproducts like lactate influence T-cell migration, with CD4^+^ and CD8^+^ T-cells showing differential lactate sensitivity due to transporter expression [346]. Lactate impairs T-cell motility through selective transporters SLC5A12 and SLC16A1, with CD4^+^ T-cells more affected by glycolysis interference through CXCR3 and CXCL10 (Figure 4). At variance, the lactic acid effect on CD8^+^ T-cell motility has been reported as more independent of glycolysis [344,350]. Tregs, however, may utilise lactate for a metabolic advantage, enhancing their infiltration over conventional T-cells [351]. Recent findings suggest that CD8^+^ T-cell motility primarily relies on the tricarboxylic acid (TCA) cycle, fuelled by glucose and glutamine, with minimal dependence on FAO and glycolysis (Figure 4) [341]. Mitochondrial dynamics influence T-cell migration. Enhancing mitochondrial metabolism has emerged as a strategy to improve CAR T-cell infiltration [352,353,354]. Elongated mitochondria promote OXPHOS, while fragmented mitochondria favour glycolysis and rapid proliferation [355]. Effector T-cells mostly depend on glycolysis, while memory T-cells rely on FAO and OXPHOS [355,356]. Memory T-cell ability to reach tissues and establish residency depends on their capacity to transmigrate and extravasate from blood vessels. During migration, T-cells polarise their mitochondria at the uropod to fuel the ATP-demanding myosin II motor [341,357].

These findings underscore the crucial role of mitochondrial dynamics in T-cell differentiation, function, and migration, with significant therapeutic implications.

### 2.8. Direct Impact of Tumour Features on T-Cell Intra-Tumour Infiltration

Tumour cells engage in many strategies to control T-cell infiltration and evade immune recognition, including modifying gene expression and activating IC pathways. For instance, tumour cells with high FAT2 expression have been reported to upregulate several chemokines that influence immune cell behaviour, including CCL2, CCL3, CCL4, CCL19, CXCL10, and CXCL11 [358].

Single-cell RNA sequencing has helped identify different tumour cell subtypes, revealing divergent effects on T-cell infiltration. Notably, a mesenchymal-like tumour state has been reported to boost T-cell cytotoxicity in glioma models [359], while a negative correlation exists between the abundance of infiltrating T-cells and the expression of genes related to EMT in colon cancer [360] and NSCLC [361].

Tumours can alter T-cell infiltration by upregulating specific genes. For example, a study has provided a high-resolution landscape of ICI-resistant genes, reporting that p53 and MYC promote T-cell exclusion in melanoma, while HLA-A and c-JUN encourage T-cell infiltration [362]. Notably, TGF-β contributes to immunosuppression through the downregulation of CD8^+^ T-cell expression of CXCR3, limiting trafficking to the tumour site (Figure 2) [363]. Mechanisms of immune evasion mediated by p53 mutation or loss include the downregulation of MHC Class I and II molecules, loss of TRAIL receptors, and upregulation of PD-L1 expression [364]. MYC overexpression further drives immune evasion by restricting neoantigen presentation, upregulating PD-L1, and metabolic reprogramming of cancer cells [365]. In contrast, HLA-A enhances tumour immunogenicity and T-cell recognition [366], while c-JUN promotes the establishment of an inflammatory signalling, favouring immune infiltration [367].

## 3. Therapeutic Strategies

### 3.1. Therapeutic Strategies to Contrast Tumour Cell Motility

Targeting key components of the TME has become a promising strategy to inhibit EMT and metastasis. Therapies have been developed against immune cells, stromal cells, vasculature, and the ECM using monoclonal Abs (mAbs) small molecules, peptides, nanoparticles, and hybrid systems. Stromal targeting, especially of CAFs, is gaining interest [368,369]. CAFs promote tumour progression by secreting ECM components, enzymes, cytokines, chemokines, and growth factors [370,371] while modulating T-cell responses and angiogenesis via TGF-β, IL-6, CXCL9, and VEGF [372]. Most therapies focus on Fibroblas Activation protein (FAP), overexpressed on CAFs [373]. RO6874281 and sibrotuzumab (NCT02627274, NCT02198274, NCT03386721) showed safety but limited efficacy [374,375]. A DNA vaccine targeting FAP reduced tumour growth in mice [376].

CAF functions rely on TGF-β, CXCR4, FGFR, PDGFR, ROCK, and NF-κB, which are under clinical investigation [377,378]. Markers like FSP1, ITGA11, and ITGB1 are being studied as prognostic tools [379]. Erdafitinib (FGFR inhibitor) is FDA-approved for FGFR-mutated urothelial cancer [380]. FGFR and Hedgehog inhibitors like vismodegib are approved for basal cel carcinoma (BCC) [381], although saridegib with gemcitabine was ineffective in pancreatic cancer (NCT01130142). Axitinib, a PDGFR inhibitor, showed benefit in metastatic RCC (NCT02684006, last accessed 31 March 2025) [382].

CAF normalisation via vitamins A or D enhances anticancer response in preclinical models [383]. Combination therapies, e.g., galunisertib with sorafenib or chemoradio-therapy, improved outcomes (NCT01246986, NCT02688712) [384,385]. CXCR4 inhibition with AMD3100 and G-CSF supports stem cell mobilisation in Multiple Myeloma (MM) and non-Hodgkin lymphoma (NHL) [386,387]. The ROCK inhibitor AT13148 showed good tolerability (NCT01585701) [388], and ATRA with apatinib was effective in metastatic adenoid cystic carcinoma (ACC) (NCT04433169) [389] and is under study in acute promyelocytic leukemia (APL) (NCT00482833) [390].

Extracellular vesicles (ECVs)—microvesicles, exosomes, and apoptotic bodies—have diagnostic and therapeutic potential [391,392,393,394]. Exosome-targeting strategies to block EMT in various cancer models include proton pump inhibitors [395], Rab27a silencing [396,397], and heparin to inhibit uptake [398], although clinical data are limited.

TAM targeting aims to restore immune surveillance [399,400,401]. CCL2 promotes TAM accumulation and metastasis [153,402]. Anti-CCL2 (carlumab) and anti-CCR2 (PF-04136309) showed limited efficacy alone, but combinations with chemotherapy yielded better outcomes (NCT01204996) [403,404,405,406].

CSF1R, essential for TAM survival, is targeted to deplete or reprogram TAMs [407,408,409,410,411,412,413,414]. Combining CSF1R inhibitors with radiotherapy or TKIs enhances efficacy [415,416,417,418,419,420,421]. Several agents (emactuzumab, PLX7486, BLZ945, edicotinib, ARRY-382, pexidartinib) are in trials [422,423]; however, pexidartinib failed in glioblastoma (NCT01349036) [424].

CD40 agonists reprogram TAMs and enhance T-cell infiltration. Agents like CP-870,893 and sotigalimab showed early efficacy [425,426,427,428]. The results vary with some trials reporting durable responses [429], while others like those employing dacetuzumab (NCT00435916) did not. CD40 also promotes endothelial activation (e.g., through induction of ICAM-1, VCAM-1, and selectin expression) [430]. The viral delivery of CD40L activates immune cells in pancreatic preclinical models [431]. Combined with sunitinib, CD40 agonists improve tumour regression through activation of DCs and MDSC reduction [432].

TREM2 is another TAM target. PY314 showed safety in Phase I trials (NCT04691375, last accessed 31 March 2025) [433]. PI3Kγ inhibition by eganelisib demonstrated efficacy with ICIs in solid tumours (NCT03961698, last accessed 31 March 2025) [434,435].

ECM modulation, particularly employing hyaluronidases and collagenases, has been reported to enhance drug delivery [436]. PEGPH20 improved PFS in pancreatic cancer [437], but subsequent trials (NCT02715804, NCT01959139) did not observe clinical benefit and notable toxicity in pancreatic cancer [438]. ECM synthesis inhibitors target HIF-1α, TGF-β, and LOX. Anti-LOXL2 Ab simtuzumab failed in clinical trials (NCT01472198, NCT01479465) [439,440].

Repurposed agents like losartan, pirfenidone, and metformin are under evaluation. FOLFIRINOX plus losartan improved outcomes in pancreatic cancer [441], while metformin showed no benefit in a Phase III breast cancer trial (NCT01101438) [442].

Integrins and FAK are also therapeutic targets [443,444]. Despite encouraging preclinical findings, integrin inhibitors still lack clinical validation [445]. The Ab– antimitotic drug conjugate SGN-B6A, targeting integrin β6, is under clinical investigation for advanced malignancies (NCT04389632) [446]. Another promising approach involves MMG49-CAR T-cells (OPC-415), designed to target the active form of integrin β7 in multiple myeloma, now in a Phase II trial (NCT04649073) [447]. FAK inhibitor defactinib is also under investigation, showing mixed results in advanced solid tumours [448,449]. These findings underscore the complexities of ECM-targeting strategies, requiring further research to improve their clinical utility.

### 3.2. Potential Approaches to Increase Intra-Tumour T-Cell Motility

A deeper understanding of chemokines (e.g., CXCL13, CXCL16, CCL5, and CCL22) and cytokines (e.g., IL-2, IL-7, and IL-15) is essential to reprogramming the TME from immunologically “cold” to “hot”, thereby enhancing immune infiltration and activity.

Several approaches have been explored to manipulate chemokine signalling. CD8^+^ T-cells, Th1 cells, and NK cells are critical for anti-tumour immunity, often expressing CXCR3. The presence of CXCR3 on circulating T-cells, along with intra-tumoural CXCL9 and CXCL10 expression, is associated with increased T-cell infiltration and improved patient survival across cancers [215,221]. This axis is primarily driven by IFN-γ [215,225,226,227], and its activation via intra-tumoural IFN-γ delivery increases CXCL10/11 levels in melanoma [450]. Preclinical administration of CXCL9/10 enhances T-cell recruitment and suppresses tumour growth [451], although clinical translation remains challenging.

ICB indirectly increases CXCR3 ligands by promoting IFN-γ from activated T-cells. PD-1 blockade increases tumoural CXCL10 in ACT-treated melanoma [452], while ipilimumab elevates CXCL9–11 levels [453]. CXCL9 from CD103^+^ DCs facilitates CXCR3^+^ T-cell recruitment in the TME [453]. IL-7 delivery and COX-2 inhibitors also promote an increase in the levels of CXCR£ ligands CXCL9/10 [453,454].

PARP inhibitors enhance intra-tumoural CD4^+^ and CD8^+^ T-cell infiltration [455,456,457]. BRCA1/2-deficient cancers show greater T-cell presence and survival [458]. Olaparib promotes CD8^+^ T-cell infiltration via the cGAS/STING pathway, especially in BRCA1-deficient tumours [459,460], while increased cytosolic DNA leads to CCL5/CXCL10-driven T-cell recruitment, independent of BRCA status [461]. However, STING agonists may benefit BRCA wild-type tumours [459].

DPP4 (CD26) inhibition elevates CXCR3 ligands, enhances CXCR3^+^ NK/T cell infiltration, and improves responses in melanoma, glioblastoma, and colorectal cancers [215,223]. Interestingly, CD26^+^ T-cells also drive tumour immunity and may predict response to PD-1 blockade [462,463,464], underscoring the dual role of CD26 in immuno-regulation.

The CCL5/CCR5 axis promotes tumour progression via survival, proliferation, MMP activation, and immune suppression through Treg, MDSC, and TAM recruitment [243]. CCR5 blockade repolarises TAMs and improves tumour control [240], although its dual role in both tumour progression [241] and effector T-cell recruitment [242] requires context-specific targeting.

GPR182, a scavenger of CXCL9/10, impairs lymphocyte recruitment and contributes to immunotherapy resistance, representing a promising target for cold tumours [465].

CAR-T therapies have transformed hematologic malignancy treatment and are advancing in solid tumours, where poor infiltration remains a challenge [466,467]. Chemokine receptor engineering (e.g., CXCR3, CXCR4) improves CAR-T migration and efficacy [468]. Co-expression of CCR5 and IL-12 in mesothelin-targeted CAR-T enhances infiltration and functionality [469]. Trials are underway for CAR-T cells expressing CXCR5 (NCT04153799, NCT05060796), CXCR2 (NCT05353530, NCT01740557), and CCR4 (NCT03602157) in various cancers [470] (https://clinicaltrials.gov, last accessed 31 March 2025). Other strategies include IL-7/CCL19-secreting CAR-T cells (NCT04381741, NCT04833504).

However, CXCR4–CXCL12 engagement supports angiogenesis, tumour survival, and immune suppression [280]. CXCR4 inhibition with AMD3100 enhances effector T-cell infiltration and reduces Tregs, promoting M1 macrophage polarisation [471].

Tumour metabolism, particularly elevated lactate levels and low pH in the TME, im-pairs CD8^+^ T-cell function via transporters MCT-1 and SLC5A12 [346,472,473]. Targeting lactate metabolism through MCT1 inhibitor AZD3965 (Phase I trials) [474], CPI-613 (targets TCA enzymes) [475], lactate oxidase nanocapsules [476], and dichloroacetate (DCA) [477] may restore T-cell functionality and bolster immune responses.

GPR65, a proton-sensing receptor, modulates T-cells in acidic TMEs. Its inhibition improves macrophage function and tumour immunity [478].

Aberrant vasculature in tumours impairs T-cell trafficking. VEGF blockade enhances vascular structure and chemokine expression, improving survival, particularly with ICB [479]. Low-dose VEGF inhibition (e.g., bevacizumab) normalises vessels and reduces pressure, facilitating T-cell entry [326,480]. HEV-like vasculature expressing PNAds and CCL21 also supports naïve T-cell infiltration, correlating with improved prognosis in melanoma, lung, and ovarian cancers [481,482]. LTα3 and IFN-γ promote HEV formation, and their localised delivery or VEGF inhibition may enhance this process. LIGHT, a TNF superfamily member, similarly promotes HEV and TLS formation while improving adhesion molecule expression and vessel normalisation [483,484,485].

Inhibiting endothelial factors (e.g., FASL) increases CD8^+^ infiltration and suppresses tumour growth, revealing an endothelial immune barrier and offering a therapeutic avenue for improving anti-tumour immunity [480].

## 4. Unveiling the Dynamic Interplay of Tumour and T-Cell Motility in the TME

The plasticity of tumour cell motility enables cancer cells to switch epithelial to mesenchymal and amoeboid phenotypes in response to cues from the extracellular matrix and immune cells [486]. This tumor dissemination relies on dynamic interactions with the immune microenvironment, where cancer cell motility and immune-regulated chemotactic and adhesion signals shape immune cell infiltration [487]. Tumour spread is a dynamic process [1], governed by intricate interactions between cancer cells and the TME, particularly immune components. The plasticity of tumour cell motility enables cancer cells to switch epithelial to mesenchymal and amoeboid phenotypes in response to cues from the extracellular matrix and immune cells [486]. 

Simultaneously, chemokines such as CXCL9, CXCL10, and CCL5 regulate T-cell recruitment [215], whereas LFA-1 and adhesion molecules like ICAM-1 [487] govern their infiltration and retention within the tumour. 

The TME shapes not only tumour cell motility and metastatic dissemination but also profoundly affects immune cell recruitment and functionality, particularly impacting T-cell trafficking. Tumour-derived factors such as hypoxia, ECM remodelling, CAFs, TAMs, and metabolic adaptations exert a dynamic influence on these processes. Hypoxia represents a central driver of both tumour cell migration and immune modulation within the TME, fostering tumour proliferation, EMT, and metastasis [92] while creating a metabolically hostile and immunosuppressive environment that hampers T-cell infiltration. The hypoxia-induced acidic milieu resulting from increased glycolysis suppresses immune cell motility and activity, promoting protease activation and matrix degradation, which in turn facilitate tumour invasion [197,198,199]. These tumour-derived adaptations remodel the TME biochemically and physically, forming barriers that T-cells must overcome to infiltrate tumours.

CAFs, activated by tumour-derived signals, are major players in the TME and contribute to the exclusion and dysfunction of T-cells. By producing TGF-β, IL-6, CXCL12, and MMPs [103,104,105,106], CAFs induce EMT and modify the ECM, enhancing tumour invasiveness while creating a stiffened, collagen-rich matrix [116,117,118,119]. Such matrix alterations not only support tumour cell motility but also generate a dense physical barrier that impedes T-cell entry and restricts their motility within tumours. In addition, CXCL12, produced by either CAFs and tumours [105,281], misdirect CTLs to the stromal area, preventing their effective targeting and elimination of cancer cells [210]. TAMs also play a critical role at the intersection between tumour progression and immune suppression. Recruited by tumour-derived chemokines such as CCL2, CCL5, and CCL8 [153,154,155], TAMs are reprogrammed to enhance EMT, tumour motility, and metastasis, while hampering T-cell infiltration. Moreover, their ECM remodelling activity [176,177,178,179] facilitates tumour dissemination but restricts T-cell penetration by altering matrix stiffness [174,175]. Furthermore, TAMs orchestrate the formation of TMEM doorways in collaboration with the tumour and endothelial cells, guided by VEGFA signalling [184,185], promoting tumour cell intravasation but also creating vascular niches potentially less accessible to T-cells. Together, these tumour-derived features generate a dynamic TME that selectively promotes tumour motility and invasion while simultaneously establishing physical, chemical, and immunological barriers that inhibit T-cell recruitment and motility. A key aspect of this interaction is how motile cancer cells and T-cells reciprocally influence each other’s motility, an important consideration for developing novel therapeutic strategies [488]. Emerging evidence shows that motile cancer cells can impact T-cell recruitment and motility by actively reshaping the TME, establishing physical and biochemical barriers that hinder T-cell infiltration and function. Through ECM remodelling, invasive tumour cells increase matrix density and fibre alignment, which not only facilitates tumour dissemination but also obstructs T-cell migration toward the tumour core [489].

The efficiency of cancer cell motility is influenced by ECM properties and cellular energetics. For instance, optimal collagen density reduces energy demands, enabling more efficient cancer cell movement [490]. However, this same efficiency contributes to nutrient competition within the TME, as highly migratory cancer cells exhibit elevated metabolic activity and consume key resources such as glucose and oxygen. This metabolic competition depletes the energy reserves necessary for T-cell function, ultimately impairing T-cell motility [491]. This dynamic results in a remodelled, immune-excluded TME, where tumour and immune cells compete for space. Spatial reorganisation driven by tumour cell movement can physically displace T-cells. A mathematical model illustrated by Marzban et al. supports this hypothesis, demonstrating that cancer–ECM interactions can generate T-cell-depleted regions, thereby compromising immune surveillance [492].

Moreover, motile cancer cells actively recruit immunosuppressive populations, TAMs and MDSCs, to invasive sites [493]. These cells secrete suppressive factors such as TGF-β, which downregulates CXCR3 expression on T-cells, further limiting their recruitment and infiltration [363]. Tumour-derived lactate accumulation and diminished adhesion signalling also reduce T-cell motility within the TME [341]. Computational models highlight that enhanced tumour cell motility increases spatial heterogeneity in adhesion landscapes, collectively impairing T-cell migration and cytotoxic efficiency [494]. Additionally, tumours degrade key chemokines essential for T-cell recruitment. Motile tumour cells frequently overexpress MMPs, such as MMP-8 and MMP-9, which cleave chemokines like CXCL9 and CXCL10, disrupting chemotactic gradients and diminishing T-cell infiltration [495]. Epigenetic mechanisms, including EZH2-mediated histone methylation and DNMT1-driven DNA methylation, further suppress CXCL9/10 expression. Inhibiting these pathways restores chemokine production, enhancing T-cell recruitment and improving immunotherapy efficacy [496]. Moreover, tumour-derived nitric oxide similarly downregulates CXCL10, impairing T-cell migration [497].

Conversely, T-cell motility can potentially exert substantial effect on tumour cell behaviour. Efficient T-cell infiltration into tumours is associated with reduced tumour cell migration and invasiveness. This is partly due to the cytotoxic elimination of migratory tumour cells and the modulation of the TME by activated T-cells [209]. Interestingly, tumour-associated EMT can suppress IFN-γ signalling [498]. On the other hand, IFN-γ released by cytotoxic T-cells can exert dual effects. It may promote EMT and metastasis by inducing ICAM1 and CXCR4 or alternatively inhibit metastasis through the upregulation of FN1 expression. Notably, low doses of IFN-γ have been linked to the acquisition of metastatic features, whereas higher doses promote tumour regression [499].

Motile T-cells within the tumour core also potentially disrupt pro-invasive ECM remodelling by releasing MMPs and lysosomal enzymes that degrade ECM components such as collagen and fibrin. This degradation impairs tumour cell survival and migration [317]. At variance, recent evidence suggests that cancer cell actin cytoskeleton undergoes extensive remodelling during the interaction with cytotoxic T-cells, altering the stability of IS and CTL motility [500].

Advanced methodologies, including live imaging [501], computational modelling [492], and single-cell communication analysis tools like CellChat [502], are shedding light on these complex, bidirectional interactions. These tools reveal how robust T-cell infiltration and motility can actively restrain tumour front expansion and suppress metastatic spread, offering novel insights into therapeutic opportunities.

## 5. Conclusions

Tumour cell motility and immune cell trafficking are tightly interconnected processes that shape both tumour progression and immune evasion. By remodelling the TME, cancer cells create physical, biochemical, and metabolic barriers that block T-cell infiltration. Conversely, effective T-cell mobilisation can restrain tumour invasion and spread. Future therapies should aim to reprogram the TME by restoring chemokine gradients, normalising the EM, and overcoming metabolic suppression to improve T-cell access and function. Integrating spatial imaging, computational modelling, and targeted interventions will be critical to not only prevent metastasis but also remodel the tumour–immune interface for lasting clinical responses. Understanding and manipulating the reciprocal regulation of tumour and immune cell motility is critical to identify predictive biomarkers and transforming immune-excluded (“cold”) tumours into immune-active (“hot”) ones for enhancing the efficacy, personalisation, and durability of cancer immunotherapy.
